# Smoke-Free Policies in the World’s 50 Busiest Airports — August 2017

**DOI:** 10.15585/mmwr.mm6646a1

**Published:** 2017-11-24

**Authors:** Michael A. Tynan, Elizabeth Reimels, Jennifer Tucker, Brian A. King

**Affiliations:** 1 Office on Smoking and Health, National Center for Chronic Disease Prevention and Health Promotion, CDC.

Exposure to secondhand smoke from burning tobacco products causes premature death and disease, including coronary heart disease, stroke, and lung cancer among nonsmoking adults and sudden infant death syndrome, acute respiratory infections, middle ear disease, exacerbated asthma, respiratory symptoms, and decreased lung function in children (*1*,*2*). The U.S. Surgeon General has concluded that there is no risk-free level of exposure to secondhand smoke (*1*). Previous CDC reports on airport smoke-free policies found that most large-hub airports in the United States prohibit smoking (*3*); however, the extent of smoke-free policies at airports globally has not been assessed. CDC assessed smoke-free policies at the world’s 50 busiest airports (airports with the highest number of passengers traveling through an airport in a year) as of August 2017; approximately 2.7 billion travelers pass through these 50 airports each year (*4*). Among these airports, 23 (46%) completely prohibit smoking indoors, including five of the 10 busiest airports. The remaining 27 airports continue to allow smoking in designated smoking areas. Designated or ventilated smoking areas can cause involuntary secondhand smoke exposure among nonsmoking travelers and airport employees. Smoke-free policies at the national, city, or airport authority levels can protect employees and travelers from secondhand smoke inside airports.

The 50 busiest airports were identified using data from the Airport International Council, which lists airports based on total passenger traffic for 2016 (*4*). The Airport International Council defines passenger traffic as the sum of enplaned passengers, deplaned passengers, and direct-transit passengers. To determine the extent of smoke-free policies at each of the 50 busiest airports worldwide, CDC reviewed and analyzed public information available on airport websites regarding availability of designated indoor smoking rooms at airports as of August 2017. Results were confirmed with information on smoke-free airports maintained by Americans for Nonsmokers' Rights Foundation* and with other Internet resources, including information intended to assist smokers in finding places where smoking is permitted in airports. In a limited number of instances where airport websites contained unclear or ambiguous statements about policies, additional information was collected from other sources, including airport personnel and local public health personnel.

Airports were considered to have a smoke-free policy if they completely prohibit smoking in all indoor areas. Airports were considered to have no smoke-free policy if they allowed smoking in any indoor areas, including designated or ventilated indoor smoking areas. Designated smoking areas can include, but are not limited to, rooms designed for smoking tobacco; areas or rooms of restaurants or bars where smoking is allowed; and designated areas and rooms in airline clubs where smoking is allowed. Policy status was assessed overall and by global region.

Among the 50 busiest airports worldwide, 23 (46%) had a smoke-free policy (Table 1). Among the top 10 busiest airports, five had a smoke-free policy (Beijing Capital, Chicago’s O'Hare International, London’s Heathrow, Los Angeles International, and Shanghai Pudong International) and five allowed smoking in certain indoor areas (Atlanta Hartsfield Jackson International, Dubai International, Hong Kong International, Paris’s Charles de Gaulle, and Tokyo International).

**TABLE 1:** Indoor smoke-free policy status of 50 busiest airports — worldwide, August 2017

Rank*	Airport	Jurisdiction	Country	Has indoor smoke-free policy^†^	Region
1	Atlanta-Hartsfield Jackson International	Atlanta	United States	No	North America
2	Beijing Capital International Airport	Beijing	China	Yes	Asia
3	Dubai International Airport	Dubai	United Arab Emirates	No	Asia
4	Los Angeles International Airport	Los Angeles	United States	Yes	North America
5	Tokyo International Airports	Tokyo	Japan	No	Asia
6	O'Hare International Airport	Chicago	United States	Yes	North America
7	Heathrow Airport	London	United Kingdom	Yes	Europe
8	Hong Kong International Airport	Hong Kong	Hong Kong	No	Asia
9	Shanghai Pudong International Airport	Shanghai	China	Yes	Asia
10	Charles de Gaulle Airport	Paris	France	No	Europe
11	Dallas/Forth Worth International Airport	Dallas/Fort Worth	United States	Yes	North America
12	Amsterdam Airport Schiphol	Amsterdam	Netherlands	No	Europe
13	Frankfurt Airport	Frankfurt	Germany	No	Europe
14	Istanbul Ataturk Airport	Istanbul	Turkey	No	Asia
15	Guangzhou Baiyun International Airport	Guangzhou	China	No	Asia
16	John F. Kennedy International Airport	New York City	United States	Yes	North America
17	Singapore Changi Airport	Changi	Singapore	No	Asia
18	Denver International Airport	Denver	United States	No	North America
19	Seoul Incheon International Airport	Incheon	Republic of Korea	No	Asia
20	Suvarnabhumi/New Bangkok International Airport	Bangkok	Thailand	No	Asia
21	Indira Gandhi International Airport	New Delhi	India	No	Asia
22	Soekarno-Hatta International Airport	Jakarta	Indonesia	No	Asia
23	San Francisco International Airport	San Francisco	United States	Yes	North America
24	Kuala Lumpur International Airport	Sepang District	Malaysia	No	Asia
25	Madrid-Barajas Airport	Madrid	Spain	Yes	Europe
26	McCarran International Airport	Las Vegas	United States	No	North America
27	Chengdu Shuangliu International Airport	Chengdu	China	No	Asia
28	Seattle-Tacoma International Airport	Seattle	United States	Yes	North America
29	Chhatrapati Shivaji International Airport	Mumbai	India	No	Asia
30	Miami International Airport	Miami	United States	Yes	North America
31	Charlotte Douglas International Airport	Charlotte	United States	Yes	North America
32	Toronto Pearson International Airport	Toronto	Canada	Yes	North America
33	Barcelona-El Prat Airport	Barcelona	Spain	Yes	Europe
34	Phoenix Sky Harbor International Airport	Phoenix	United States	Yes	North America
35	Gatwick Airport	London	United Kingdom	Yes	Europe
36	Taiwan Taoyuan International Airport	Taipei	Taiwan	No	Asia
37	Munich Airport	Munich	Germany	No	Europe
38	Sydney International Airport	Sydney	Australia	Yes	Oceania
39	Kunming International Airport	Kunming	China	No	Asia
40	Shenzhen Bao'an International Airport	Bao'an	China	Yes	Asia
41	Orlando International Airport	Orlando	United States	Yes	North America
42	Leonardo da Vinci–Fiumicino Airport	Rome	Italy	No	Europe
43	George Bush Intercontinental Airport	Houston	United States	Yes	North America
44	Mexico City International Airport	Mexico City	Mexico	No	North America
45	Shanghai Hongqiao International Airport	Shanghai	China	Yes	Asia
46	Newark Liberty International Airport	Newark	United States	Yes	North America
47	Ninoy Aquino International Airport	Manila	Philippines	No	Asia
48	Narita International Airport	Narita	Japan	No	Asia
49	Minneapolis/St Paul International Airport	Minneapolis/St Paul	United States	Yes	North America
50	Hamad International Airport	Doha	Qatar	No	Asia

*Ranked by total 2016 passenger traffic, according to the Airports Council International.

^†^ Airports are considered to have a smoke-free policy if they completely prohibit smoking in all indoor areas. Airports were considered to have no smoke-free policy if they allowed smoking in any indoor areas, including designated or ventilated indoor smoking areas.

Regional differences were observed in smoke-free policy status among the world’s 50 busiest airports (Table 2). Among those in North America, 14 of 18 had a smoke-free policy; in Europe, four of nine had a smoke-free policy, including airports in Madrid, Barcelona, and London (Heathrow and Gatwick airports); and in Asia, four of 22 had a smoke-free policy (all four are in China, including Beijing Capital International Airport, the world’s second busiest airport). The only airport among the 50 busiest in Oceania is Sydney International, which is smoke-free. None of the world’s 50 busiest airports is located in South America or Africa.

**TABLE 2:** Smoke-free airports among the 50 busiest airports, by region — worldwide, August 2017

Region*	No. (%) of airports among 50 busiest	No. (%) of airports with indoor smoke-free policies^†^
Asia	22 (44)	4 (18)
Europe	9 (18)	4 (44)
North America	18 (36)	14 (78)
Oceania	1 (2)	1 (100)
**Total**	**50 (100)**	**23 (46)**

*No airports among the world’s 50 busiest were in the Africa or South America regions.

^†^ Airports are considered to have a smoke-free policy if they completely prohibit smoking in all indoor areas. Airports were considered to have no smoke-free policy if they allowed smoking in any indoor areas, including designated or ventilated indoor smoking areas.

## Discussion

As of August 2017, nearly half (46%) of the 50 busiest airports worldwide have a smoke-free policy. Smoke-free policies substantially improve indoor air quality and reduce secondhand smoke exposure among nonsmokers (*1*,*2*). The 2006 Surgeon General’s report concluded that eliminating smoking in indoor spaces fully protects nonsmokers from exposure to secondhand smoke, and that separating smokers from nonsmokers, cleaning the air, and ventilating buildings cannot eliminate exposure of nonsmokers to secondhand smoke (*1*).

Although the airports in this analysis that do not have smoke-free policies only allow smoking indoors in designated or ventilated smoking areas, studies have documented that secondhand smoke can transfer from designated smoking areas into nonsmoking areas in airports, where nonsmoking travelers and employees can be exposed (*5*–*7*). In addition to subjecting nonsmoking travelers who pass through these areas to involuntary secondhand smoke exposure, designated or ventilated smoking areas can also result in involuntary exposure of airport employees who are required to enter these areas or work near them. 

Since 2012, two of the five large-hub U.S. airports that allowed smoking in designated indoor areas have implemented, or are implementing, smoke-free policies. Salt Lake City International, a large-hub U.S. airport that is not among the world’s 50 busiest, closed its smoking rooms,^†^ and Denver International closed three of its four indoor smoking rooms, with the final smoking room scheduled to close by 2018.^§^

The findings in this report are subject to at least three limitations. First, information on smoke-free policies was based on information available on airport websites, which could be subject to bias or be outdated. However, these data were cross-checked with secondary information sources, and questions about unclear information were resolved by contacting local public health and airport personnel. Second, it was not possible to identify the types of smoking areas that were allowed in all airports (e.g., rooms used exclusively for smoking, smoking sections in restaurants and bars, rooms or areas in airline clubs, etc.), nor was it possible to ascertain passenger or employee movement through airports, which might or might not include use of or proximity to areas where smoking is permitted. In addition, because it was not possible to identify smoke-free policies in outdoor areas or areas near exits, this information was not reported. Finally, only the 50 busiest airports were included in this study; therefore, regions such as South America and Africa were not represented in the study because they did not include any of these busiest airports. However, many airports with lower passenger volume have implemented smoke-free policies (*8*).

Progress has been made in protecting nonsmoking passengers and employees from secondhand smoke in airports. A majority of airports are smoke-free in many countries worldwide, including Australia and New Zealand; European countries such as Denmark, Ireland, Norway, Spain, and the United Kingdom; South American countries such as Argentina, Brazil, Chile, Ecuador, and Uruguay; and North American countries such as Canada and the United States.^¶^ Smoke-free policies at the national, city, or airport authority levels can protect employees and travelers from secondhand smoke inside airports.

**Summary**

**What is already known about this topic?**

There is no risk-free level of exposure to secondhand smoke. Eliminating smoking in indoor spaces fully protects nonsmokers from exposure to secondhand smoke. An overwhelming majority of large-hub airports in the United States prohibit smoking indoors.

**What is added by this report?**

Among the 50 busiest airports worldwide, 23 airports (46%), including five of the 10 busiest airports, prohibit smoking in all indoor areas. While smoke-free airports among the 50 busiest are common in North America (14 of 18), few airports in Asia (4 of 22) have implemented smoke-free polices.

**What are the implications for public health practice?**

Broader implementation of smoke-free policies at the national, city, or airport authority levels can protect employees and travelers of all ages from secondhand smoke inside airports.

## Footnotes

*
http://no-smoke.org/learnmore.php?id=187 .

† http://www.sltrib.com/news/3928480-155/salt-lake-city-to-phase-out; https://www.slcairport.com/airport-services/smoking-areas/.

§
https://www.flydenver.com/sites/default/files/downloads/DIAPR_130111s.pdf .

¶
http://no-smoke.org/pdf/Smokefree-Airport-Highlights-From-Around-the-World.pdf .
